# Supplementary material for: Human response times are governed by dual anticipatory processes with distinct neural signatures
Source: Commun Biol. 2025 Jan 26;8:124. doi: 10.1038/s42003-025-07516-y (PMC11762298; doi:10.1038/s42003-025-07516-y)
Supplement: Supplementary file 1 — Supplemental Material [file 42003_2025_7516_MOESM1_ESM.pdf]

Supplementary Materials for

Human Response Times are Governed by Dual Anticipatory Processes with Distinct Neural Signatures

Ashwin G Ramayya, Vivek Buch, Andrew Richardson, Timothy Lucas, and Joshua I. Gold

Correspondence to: aramayya@stanford.edu

This PDF file includes:

1. Individual model fits (Fig. S1)
2. Model simulations to account for group-level behavior (Fig. S2)
3. Overview of methods used to relate each electrode's activation function to endogenous RT variability (Fig. S3)
4. Anatomical parcellation by brain region (Fig. S4, Table S1)
5. Cluster descriptions (Fig. S5)
6. Participant characteristics and behavior (Tables S2 and S3)

1. Individual model fits

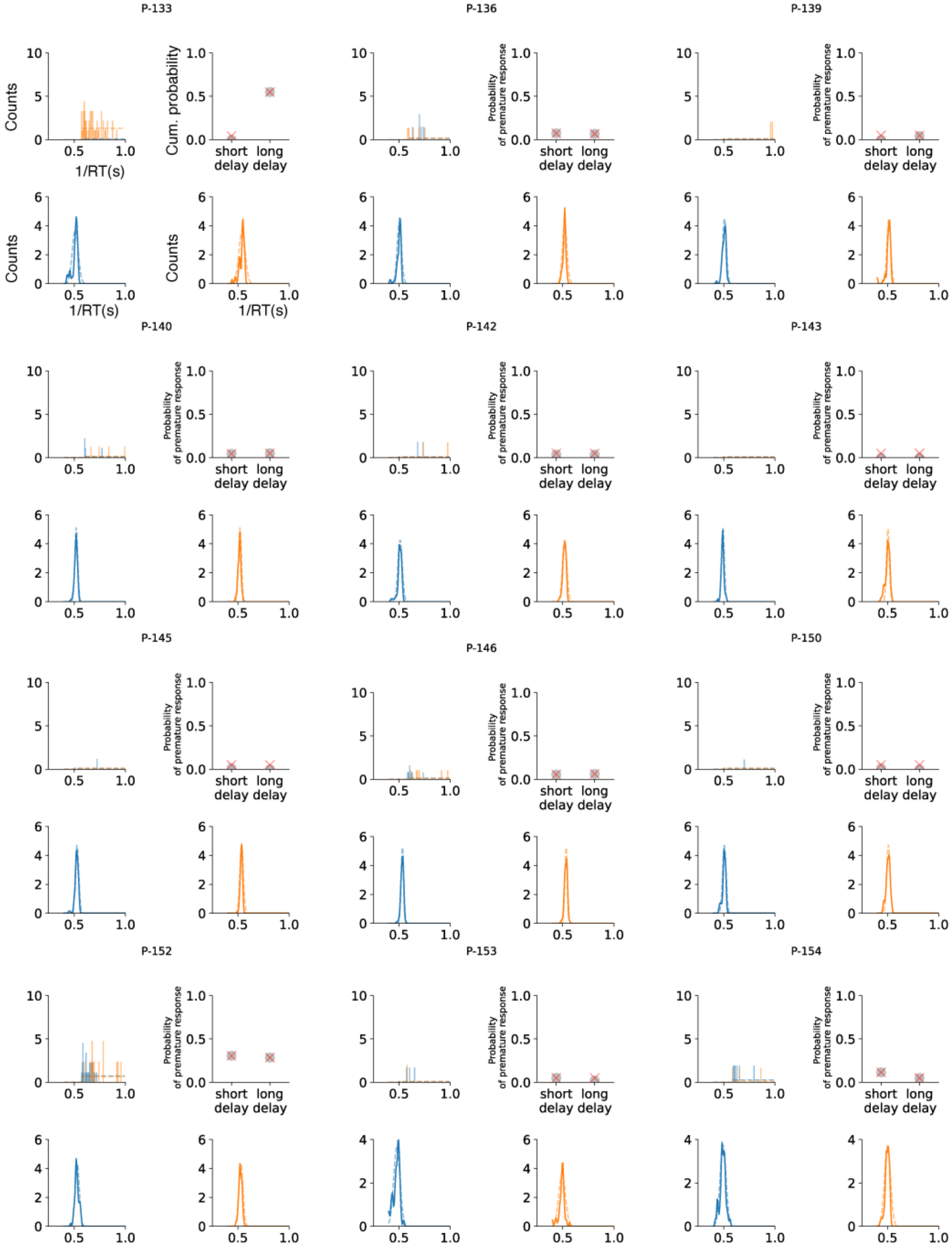

Figure S1. Individual model fits (Participant ID 133–154). Data from each participant are summarized in each block of four panels. The top-left panel shows the observed distribution of reciprocal RTs for premature false alarms during short-delay (blue vertical lines) and long-delay (orange vertical lines) trials, and the model-based uniform process underlying each distribution (dashed horizontal lines). The top-right panel shows the observed cumulative false-alarm rate for short- and long-delay trials (gray circles) and model predicted false-alarm rates (red crosses). Bottom panels show reciprocal RT distributions (solid line) and model fits (dashed line) on short-delay trials (blue, left panel) and long-delay trials (orange, right panel).

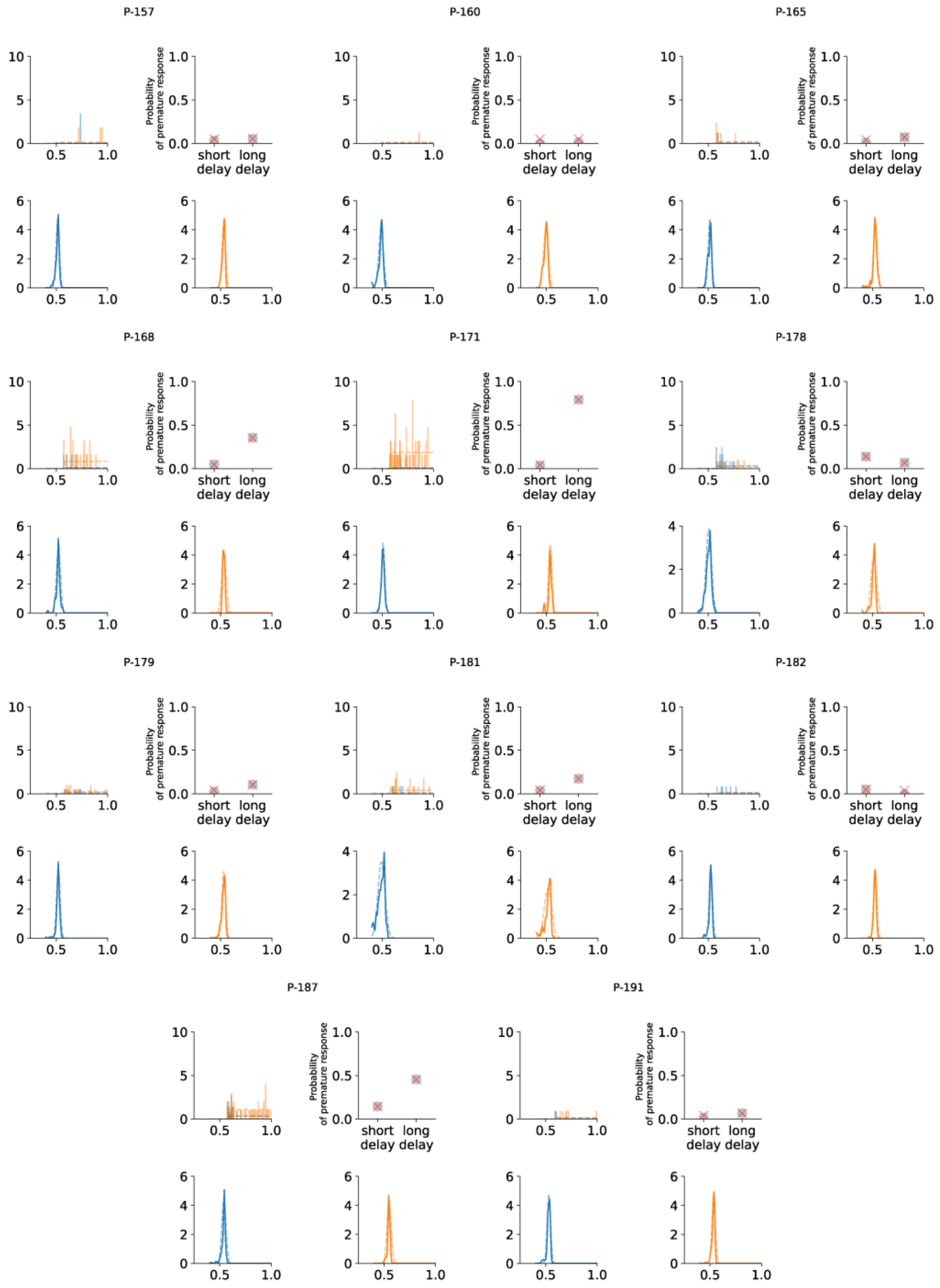

Figure S1. Individual model fits (Participant ID 157-191)

2. Model simulations to account for group-level behavior

We simulated model behavior on the variable foreperiod delay task used in this study (1000 simulated trials for each delay condition). We expanded the model presented in the manuscript to include a mechanism to resolve competition between false-alarm and correct responses, as follows. During each trial, a "prestimulus" rising process was triggered 500 ms prior to the onset of S2 by drawing a rate-of-rise from a gaussian distribution. If this rising process reached the threshold value (fixed at a value of 1) prior to the onset of S2, a false-alarm response was generated. Otherwise, a "poststimulus" rising process was triggered following the presentation of S2, which triggered a correct response if it reached the response threshold within the response interval (1 s). We established a base model to simulate behavior in the absence of anticipatory processing by identifying parameters for the starting points and rate-of-rise distributions of these sequential rising processes that produced mean RTs and FA rates that roughly matched aggregate human behavior on short-delay trials (mean RT of 458 ms; mean FA rate 0.1%). Figure S2 illustrates an observed RT distribution (Fig. S2A, reproduced from Fig. 1a) and a simulated RT distribution using this base model (Fig. S2B). The model parameters are as follows: response threshold, 1; prestimulus starting point 0; prestimulus mean rate-of-rise, 1; prestimulus std rate-of-rise, 0.2; poststimulus starting point 0; poststimulus mean rate-of-rise, 2.25; poststimulus std rate-of-rise, 0.4). Of note, we considered time in units of seconds and a fixed response threshold of 1 when determining these parameter values.

We considered these parameters to provide a model of "short-delay" RT distributions. We modeled anticipatory bias by modifying select parameters of this base model, and then using the modified parameters as a model of "long-delay" RT distributions that would be generated in the setting of increased anticipation. For example, a poststimulus starting-point bias of 0.1

corresponded to generating an RT distribution using the same parameters as the base model except a value of 0.1 for the poststimulus starting point. We evaluated each model of anticipatory bias by measuring delay-related changes in mean RT and FA rates (by comparing RT and FA rates generated by the base short-delay model and the modified long-delay model). These measures allowed us to relate model simulations to observed group-level human behavioral data (as shown in Fig. 1E). Then, we could assess how each model of anticipatory bias compared to the observed changes in mean RT and FA rate observed in the group-level behavioral data.

First, we used this approach to supplement our model comparison analysis in the main manuscript. We evaluated three distinct models of anticipatory bias in RTs (Fig. S2C): 1) increased starting-point on long-delay trials, 2) increased mean rate-of-rise distribution on long-delay trials, and 3) increased variance of rate-of-rise distribution on long-delay trials. All three of these models included increases in the starting point of the prestimulus rising process to produce false alarms (see below for more details). We modified each parameter using bias values ranging from -0.1 to 1 (100 linearly spaced values). In the figure below, each gray circle represents simulated behavioral data from a unique parameter modification. The black box indicates the range of observed changes in mean RT and FA rate observed in the group-level behavioral data (shown in Fig. 1E). We found that model 1 and model 2 had sufficient explanatory power to generate the observed behavioral data (Fig. S2D,E), but model 3 did not (Fig. S2F). This result is consistent with our findings from the model-comparison analysis presented in the main text.

Second, we evaluated our approach of using independent modulations of prestimulus and poststimulus starting points when modeling anticipatory bias (Model 1, above). This approach was motivated by the fact human participants showed only a weak correlation between delay-

related changes in RT and FA rate, suggesting that these behaviors arise from distinguishable cognitive processes. In support of this idea, we found that an alternative modeling strategy of modifying prestimulus and poststimulus starting points in a correlated manner had insufficient explanatory power to explain the full range of observed behavioral changes. In particular, simulating these modifications in a correlated manner resulted in correlated changes in mean RT and FA rates (Fig. S2I). Likewise, simulated modulations of prestimulus starting point only (Fig. S2G; increased FA rate, but no change in mean RT) or poststimulus starting point only (Fig. S2H; faster mean RT, but no increase in FA rate) yielded predictions that did not match our data. These results provide support in favor of the additional explanatory power provided by using independently varying starting points in our models.

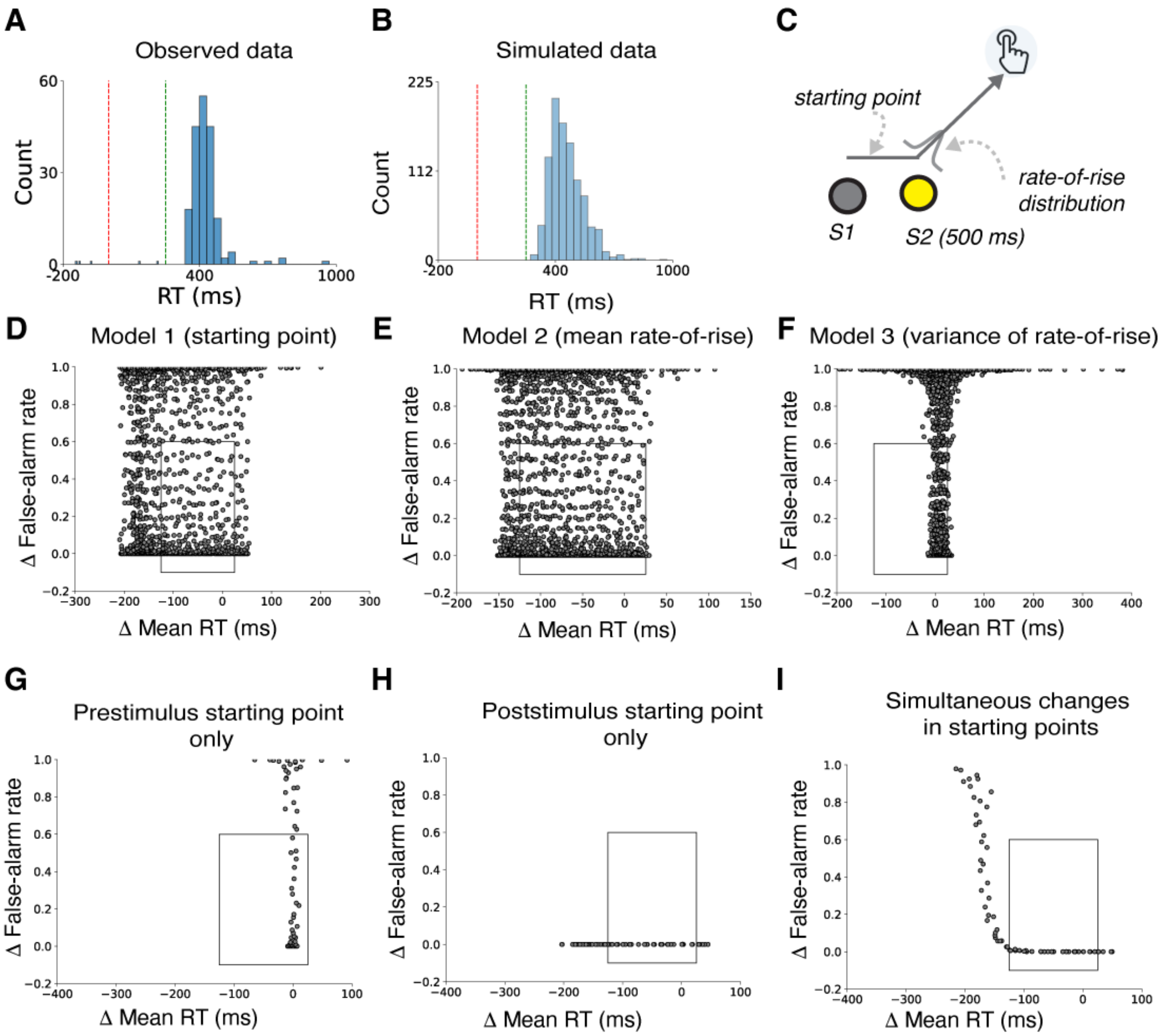

Figure S2. Model Simulations. (**A**) Example RT distribution from short-delay trials from a human participant (reproduced from Fig. 1A). (**B**) Simulated RT distribution from the base model of RT behavior on short-delay trials. (**C**) Schematic illustration of the poststimulus rise-to-bound process underlying RT distributions. (**D-I**) Model simulations of anticipatory FA bias (ordinate) and RT bias (abscissa). The black box indicates the range of observed group-level changes in mean RT and FA biases observed in human participants (as shown in Fig. 1E). Each gray circle represents simulated behavioral data from a unique parametric modification to the base model, as follows: (**D**) independent changes in prestimulus starting point and poststimulus starting point modulations (Model 1); (**E**) independent changes in prestimulus starting point and mean of poststimulus rate-of-rise (Model 2); (**F**) independent changes in prestimulus starting point and variance of poststimulus rate-of-rise (Model 3); (**G**) Isolated changes in prestimulus starting point; (**H**) Isolated changes in poststimulus starting point; (**I**) Simultaneous (correlated) changes prestimulus and poststimulus starting points.

3. **Overview of methods used to relate each electrode's activation function to endogenous RT variability**

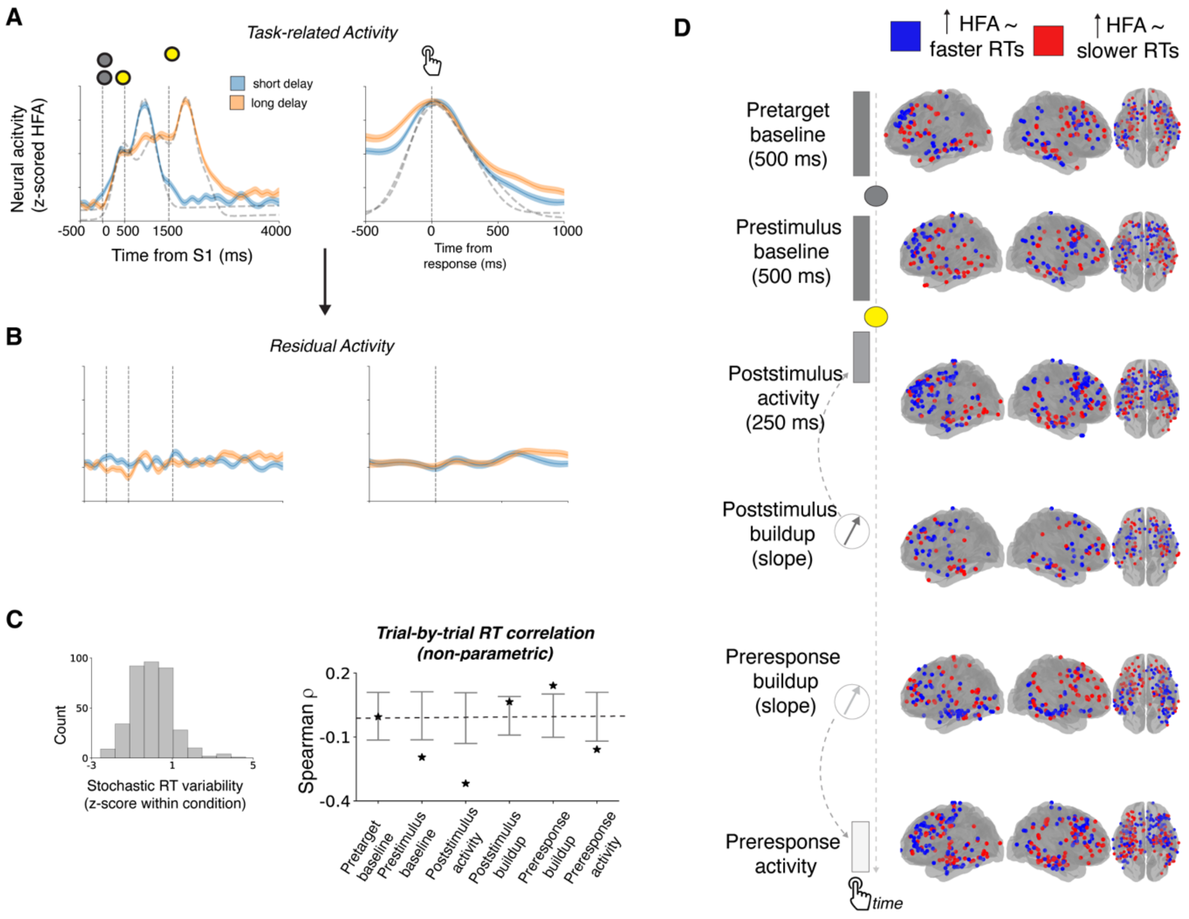

Figure S3. Overview of methods used to relate each electrode's activation function to endogenous RT variability. (**A**) Example target-, stimulus-, and response-locked activation functions from a single electrode (same electrode as in Fig. 2C, without separation by RT). (**B**) Same as in A, but with stereotypical sensory and motor responses (determined via Gaussian fits to data across trials) removed. (**C**) Left panel shows the RT distribution measured in the same session as the neural data shown in **A** and **B**, with delay-related effects removed (z-scored separately per delay condition). Right panel shows how the residual activity shown in *B* measured at different times within a trial related to this RT variability. Asterisks indicate Spearman *ρ's* computed from the data between trial-by-trial z-scored RT and neural activity, $p<0.05$; error bars indicate 95% confidence intervals. **(D)** Brain plots showing the anatomical distribution of RT-related neural representations ($p<0.05$, non-parametric tests). Blue electrodes indicate negative effects (relatively increased activity during fast RTs), red electrodes indicate positive effects (relatively increased activity during slow RTs). Each row depicts data from different task epochs, as indicated and as in **C**.

4. Anatomical parcellation by brain region

We performed additional analyses using finer-grained anatomical localization (Fig. S4). Overall, these findings were similar to those we presented using intrinsic brain networks.

Specifically, we used Advanced Normalization Tools (Avants et al., 2011) to register MRI to the Neuromorphometrics whole brain atlas (Landman & Warfield, 2012). Figure S4A shows a brain plot showing electrode locations from all subjects in standard MNI coordinates. Colors indicate Regions of Interest as shown on the right (Table S1). Electrodes were assigned anatomical labels in a hierarchical manner. First, electrodes were labeled using the Neuromorphometrics atlas. Second, for all electrodes labelled as "Cerebral White Matter," we assigned a specific white-matter tract label by relating MNI coordinates of each electrode to the XTRACT Human Connectome Project Probabilistic atlas (Warrington et al., 2020). Third, for white-matter electrodes that remained without a specific tract label, we assigned a lobe-based label using the MNI Structural Atlas (e.g., "parietal white matter" or "frontal white matter") (Mazziotta et al., 2001). For electrodes that were not assigned to an anatomical structure based on the above procedures, we assigned the label associated with the nearest labeled electrode in MNI coordinate space (using Euclidean distance). We grouped electrodes into regions of interest (ROI) based on manual grouping of anatomical labels (Table S1).

In general, we found that neural activity modulated by task events and/or showed trial-by-trial correlations with RTs (as defined in the main text) was widely distributed across brain regions of interest. Figure S4B and C show a replication of the results shown in Figs. 3B and 3D using brain regions of interest (left and right, respectively). The colors correspond to Fig. S4A.

Using the brain regions of interest shown in Fig. S4A, we did not find localized neural correlates of anticipatory biases as we did using functionally defined clusters of neural

populations (Fig. 4). We found that prestimulus activity levels showed only weak and non-significant correlations with anticipatory behavioral biases across brain regions (FDR-corrected p's > 0.5; Fig. S4E). This result also held true when testing specific subregions of interest that have been highlighted by prior functional neuroimaging studies, including: superior temporal gyrus (n=40 electrodes, 13 participants); posterior insula (n=40 electrodes, 9 participants); anterior insula (n=37; 16 participants); dorsal cingulate (n=31; 12 participants); inferior frontal gyrus (n=44 electrodes, 12 participants); and middle frontal gyrus, which overlaps with DLPFC (n=120 electrodes, 20 participants). We excluded the following regions from these analyses because they did not have sufficient data (< 5 participants): mid cingulate (n=7 electrodes, 4 participants) and supplementary motor area (n=4 electrodes, 3 participants).

In summary, finer-grained anatomical localization did not provide evidence for localized neural substrates of anticipatory biases. These results further motivate our approach of assessing correlates of anticipatory biases using clusters of functionally defined neural populations, rather than specific brain regions.

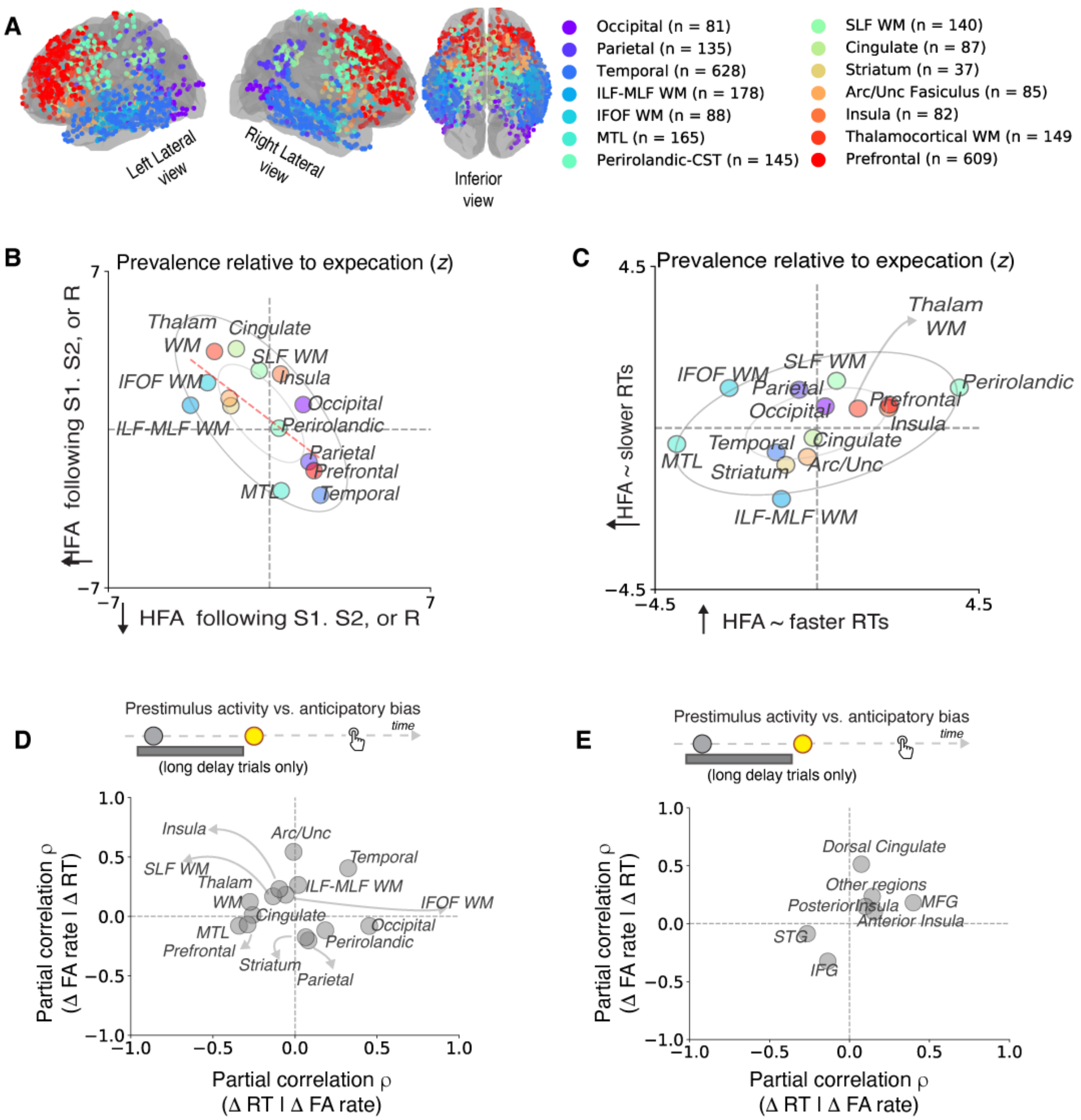

Figure S4. Anatomical parcellation with brain regions. (**A**) Brain plot showing electrode locations from all participants in standard MNI coordinates. Colors indicate brain regions of interest based on co-registration with normative atlases (Table S1). (**B**) Scatterplot showing the relative frequency of electrodes with positive (ordinate) and negative (abscissa) changes in activity to task-relevant events in each intrinsic brain network relative to their overall (expected) frequency across the brain (z-scores). Positive values indicate increased relative frequency; negative values indicate decreased relative frequency. Inner and outer ellipses indicate 1σ and 2σ confidence intervals derived from the joint distribution, respectively. (**C**) Same as **B**, but for positive and negative correlations with delay-independent, trial-to-trial RT variability during any

time interval. (**D**) The top schematic shows the time interval used to compute prestimulus activity in each region of interest. The scatterplot shows partial correlation coefficients for RT bias (controlling for FA bias) on the abscissa and FA bias (controlling for RT bias) on the ordinate. (**E**) Same as **D**, but for specific regions implicated by prior functional neuroimaging studies (Cui et al. 2009; Vallesi et al 2009).

Table S1.
Anatomical labels used to define Regions of Interest

Occipital'	MOG middle occipital gyrus', 'SOG superior occipital gyrus', 'OFuG occipital fusiform gyrus', 'IOG inferior occipital gyrus','LiG lingual gyrus','Occipital Lobe_wm','Optic Radiation L', 'Optic Radiation R','Forceps Major', 'Vertical Occipital Fasciculus L', 'Vertical Occipital Fasciculus R'
'Parietal'	AnG angular gyrus', 'PCu precuneus', 'SPL superior parietal lobule', 'SMG supramarginal gyrus', 'PO parietal operculum', 'Parietal Lobe_wm'
'Temporal'	FuG fusiform gyrus', 'ITG inferior temporal gyrus', 'PT planum temporale', 'MTG middle temporal gyrus', 'FuG fusiform gyrus/"BA36', 'PHG parahippocampal gyrus', 'STG superior temporal gyrus', 'TTG transverse temporal gyrus', 'PP planum polare', 'TMP temporal pole', 'Temporal Lobe_wm', 'Acoustic Radiation L', 'Acoustic Radiation R'
'ILF-MLF WM'	Inferior Longitudinal Fasciculus L', 'Inferior Longitudinal Fasciculus R', 'Middle Longitudinal Fasciculus L', 'Middle Longitudinal Fasciculus R'
'MTL'	Hippocampus', 'Amygdala', ' entorhinal area', 'Fornix L', 'Fornix R'
'Perirolandic-CST'	'PoG postcentral gyrus', 'CO central operculum', 'PrG precentral gyrus','Corticospinal Tract L', 'Corticospinal Tract R'
'IFOF WM'	Inferior Longitudinal Fasciculus L', 'Inferior Longitudinal Fasciculus R', 'Middle Longitudinal Fasciculus L', 'Middle Longitudinal Fasciculus R'
'SLF WM'	Superior Longitudinal Fasciculus 1 L', 'Superior Longitudinal Fasciculus 1 R', 'Superior Longitudinal Fasciculus 2 L', 'Superior Longitudinal Fasciculus 2 R', 'Superior Longitudinal Fasciculus 3 L', 'Superior Longitudinal Fasciculus 3 R'
'Cingulate'	ACgG anterior cingulate gyrus', 'PCgG posterior cingulate gyrus', 'MCgG middle cingulate gyrus', 'Cingulum subsection: Dorsal L', 'Cingulum subsection: Dorsal R', 'Cingulum subsection: Peri-genual L', 'Cingulum subsection: Peri-genual R', 'Cingulum subsection: Temporal L', 'Cingulum subsection: Temporal R'
'Striatum'	Putamen', 'Caudate_wm', 'Caudate', 'Putamen_wm'
'Arc/Unc Fasiculus'	Arcuate Fasciculus L', 'Arcuate Fasciculus R', 'Uncinate Fasciculus L', 'Uncinate Fasciculus R'
'Insula'	PIns posterior insula', 'AIns anterior insula', 'Insula_wm'
'Thalamocortical WM'	Anterior Thalamic Radiation R', 'Anterior Thalamic Radiation L', 'Superior Thalamic Radiation L', 'Superior Thalamic Radiation R', 'Frontal Aslant Tract L', 'Frontal Aslant Tract R'
'Prefrontal'	FO frontal operculum', 'OpIFG opercular part of the inferior frontal gyrus', 'OrIFG orbital part of the inferior frontal gyrus', 'MFG middle frontal gyrus', 'TrIFG triangular part of the inferior frontal gyrus', 'SMC supplementary motor cortex', 'POrG posterior orbital gyrus', 'SFG superior frontal gyrus', 'GRe gyrus rectus', 'MOrG medial orbital gyrus', 'MFC medial frontal cortex', 'MSFG superior frontal gyrus medial segment', 'AOrG anterior orbital gyrus', 'FRP frontal pole', 'Forceps Minor', 'Anterior Commissure', 'Frontal Lobe_wm'

5. Cluster descriptions

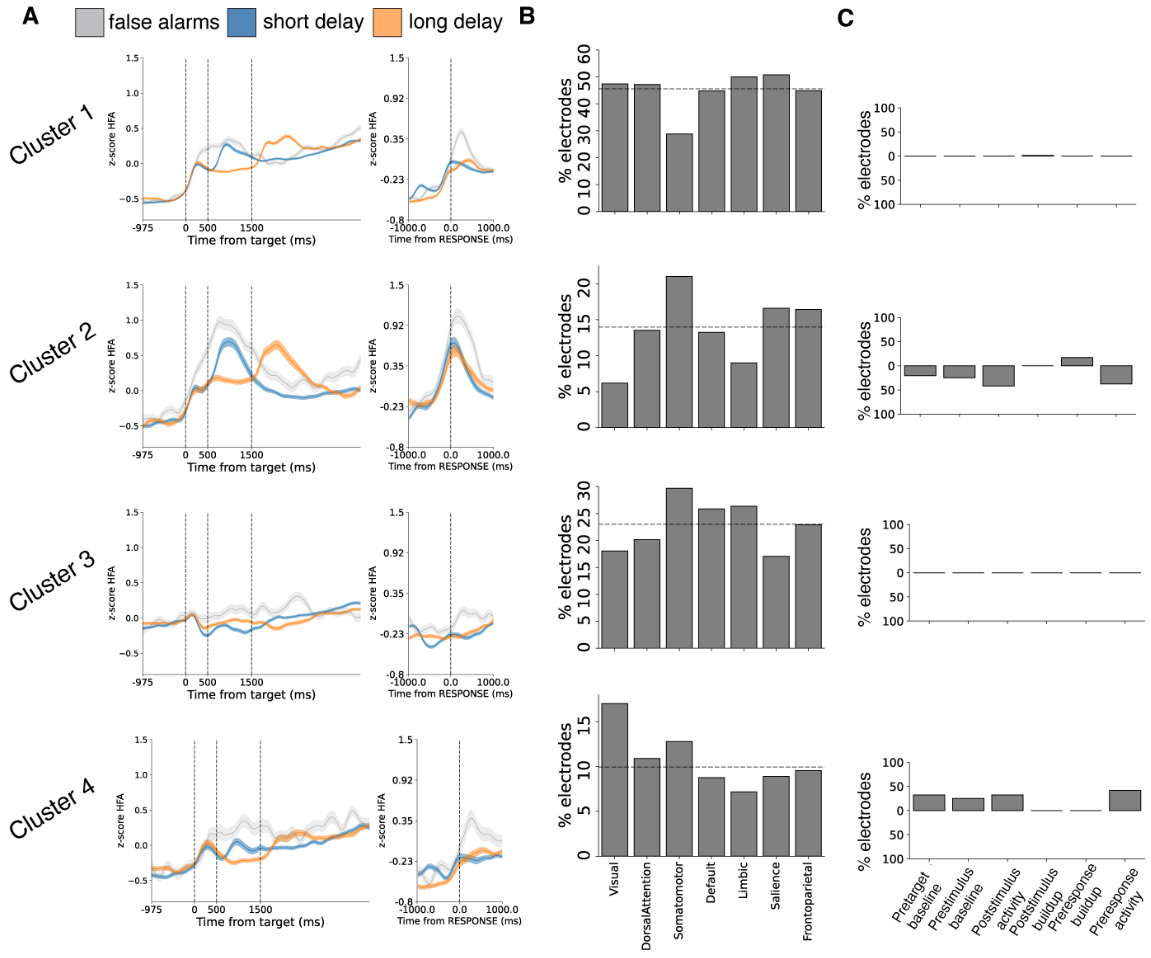

Figure S5. Cluster descriptions. Functional and anatomical properties of Clusters 1–4. (**A**) Average activation function (same format as Fig. 6B, but also including short-delay trials and response-locked activity). (**B**) Anatomical distribution of electrodes for each cluster across intrinsic brain networks. Height of bars indicate percentage of electrodes in each intrinsic brain network assigned to the given cluster. Horizontal dashed line is the expected percentage, assuming a uniform anatomical distribution across all networks. (**C**) Bar plot showing frequency of RT modulations for various task epochs (as illustrated in Figs. S2 and 4). Positive and negative values indicate frequency of electrodes showing positive effects and negative effects, respectively (as described in Fig. 3)

6. Participant characteristics and behavior

Table S2. Participant characteristics

Participant ID	Gender	Age at implant	Number of electrodes
133	Female	52	51
136	Female	57	103
140	Female	48	39
142	Male	30	68
143	Female	30	97
145	Male	21	96
146	Male	19	96
150	Male	16	90
152	Female	33	79
153	Female	56	92
154	Female	43	199
157	Male	25	145
160	Female	46	148
165	Female	20	93
168	Male	27	170
171	Male	49	160
178	Female	38	140
179	Female	19	140
181	Female	31	138
182	Female	25	118
184	Male	22	161
187	Male	24	74
191	Female	32	112

Table S3. Participant Behavior

Participant	n. trials	n. sessions	mean RT on long delay (ms)	std RT on long delay (ms)	mean RT on short delay (ms)	std RT on short delay (ms)	false alarm rate on short delay	false alarm rate on long delay	lapse rate on short delay	lapse rate on long delay
133	248	2	352.32	100.83	465.22	122.59	0.00	0.28	0.03	0.01
136	236	1	425.99	53.87	489.51	95.57	0.06	0.08	0.00	0.03
139	119	1	455.97	103.75	475.06	76.10	0.00	0.07	0.05	0.07
140	177	1	428.92	52.22	422.20	53.46	0.01	0.15	0.00	0.00
142	115	1	425.36	59.68	460.05	87.90	0.04	0.03	0.02	0.03
143	110	1	487.45	92.09	531.96	56.92	0.00	0.02	0.02	0.00
145	167	1	364.29	31.22	396.15	64.73	0.01	0.05	0.01	0.00
146	234	1	375.09	50.71	373.64	46.95	0.01	0.16	0.01	0.00
150	167	1	467.87	76.21	479.84	68.42	0.01	0.05	0.00	0.00
152	164	1	398.42	53.55	389.61	72.92	0.08	0.57	0.01	0.00
153	124	1	529.43	123.42	595.26	141.85	0.00	0.12	0.11	0.05
154	120	1	501.74	90.12	533.73	122.79	0.02	0.10	0.02	0.01
157	116	1	379.88	46.81	444.45	65.56	0.03	0.09	0.00	0.02
160	174	1	523.43	81.61	547.32	103.38	0.00	0.05	0.09	0.01
165	166	1	400.61	95.09	435.12	63.88	0.00	0.05	0.00	0.00
168	130	1	373.69	28.14	422.86	82.53	0.00	0.28	0.00	0.00
171	157	1	366.53	72.75	464.91	62.67	0.02	0.59	0.01	0.00
178	260	2	451.93	91.35	478.19	110.55	0.02	0.20	0.05	0.05
179	403	3	390.27	79.01	423.30	72.34	0.02	0.09	0.01	0.01
181	263	2	451.22	139.21	539.17	152.55	0.02	0.17	0.11	0.05
182	225	2	399.13	43.01	402.90	61.81	0.03	0.05	0.00	0.01
187	290	2	323.76	40.88	354.84	77.84	0.00	0.49	0.00	0.01
191	231	2	372.18	50.44	386.12	63.19	0.00	0.12	0.00	0.00
